# Self-Limited Epilepsy With Autonomic Seizures: A Case Report

**DOI:** 10.7759/cureus.66355

**Published:** 2024-08-07

**Authors:** Armando Felgueiras, Sérgio Bolas, Diogo M Ferreira, Dina Raimundo

**Affiliations:** 1 Family Medicine, USF (Unidade de Saúde Familiar) Cuidar Saúde, Seixal, PRT; 2 Pediatric Medicine, Unidade Local de Saúde da Região de Aveiro, Aveiro, PRT

**Keywords:** self-limited epilepsy with autonomic seizures, language disorder, epileptic syndromes, neurodevelopment, childhood epilepsy

## Abstract

This case report describes a six-year-old girl without relevant personal or family history, who had a seizure at awakening with loss of muscle tone, sialorrhea and ocular retroversion. The episode lasted >5 minutes, with vomiting and post-ictal confusion. Upon the hospital visit, she was misdiagnosed with acute gastroenteritis and discharged with symptomatic treatment.

After another seizure, she was diagnosed with Panayiotopoulos syndrome and started receiving treatment. Since then, the child has been followed up through neuropediatric appointments and by her family doctor. The psychological assessment revealed normal general intellectual functioning with vulnerability in the language area.

## Introduction

Epileptic syndromes are among the most common neurological diseases in childhood. Self-limited epilepsy with autonomic seizures (SeLEAS), formerly known as Panayiotopoulos syndrome (PS), occurs between three and six years and affects 13% of children who had ≥1 afebrile seizures [1].

SeLEAS is clinically characterized by focal convulsive seizures with prominent autonomic features, with vomiting being the most characteristic and frequent ictal sign. Syncope-like seizures with sudden loss of muscle tone and unresponsiveness are also very common. Most seizures are characterized by the progressive impairment of consciousness alongside staring or head and eye deviation. They can occur at any time during the day, more commonly during sleep, and last up to 30 minutes [1-3].

The ictal clinical features of SeLEAS are unusual for epileptic seizures and frequently mimic nonepileptic conditions, such as syncope, migraine, cyclic vomiting syndrome, motion sickness, sleep disorder, gastroenteritis and acute encephalitis. Thus, such cases are often misdiagnosed, causing high morbidity and costly mismanagement [4].

The diagnosis is based on suggestive clinical history in a child with typical age and characteristic electroencephalogram (EEG) findings that may reveal multifocal spikes, often with occipital predominance, mainly during sleep [3].

Education about SeLEAS nature and prognosis is the cornerstone of correct management. Specifically, parents should learn what to do during a crisis and about emergency therapy (rectal diazepam). Maintenance treatment with antiepileptic drugs (levetiracetam, oxcarbamazepine, or carbamazepine) is indicated for those children with unusually frequent seizures - they can reduce seizures' frequency and severity by decreasing excitation or enhancing inhibition, controlling abnormal electrical activity in the brain [1].

This syndrome has an excellent prognosis, as most children have adequate psychomotor and cognitive development with crises ceasing 2-3 years after the first episode. The risk of developing epilepsy in adulthood is similar to that of the general population [1,2,5].

## Case presentation

This case report discusses a six-year-old female child without any relevant personal or family history of seizures. She had a seizure at awakening lasting >5 minutes, only partially witnessed by her parents, with loss of muscle tone, sialorrhea and ocular retroversion. Additionally, post-ictal vomiting and dizziness occurred. The child was taken to the hospital, where she was misdiagnosed with acute gastroenteritis and discharged with symptomatic treatment.

**Figure 1:**
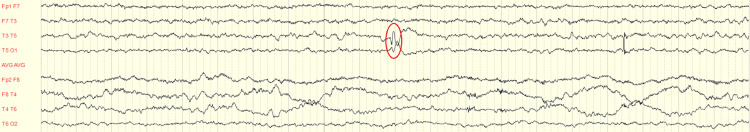
Paroxysmal activity of spike-wave accidents in the left temporal leads (T5)

**Figure 2:**
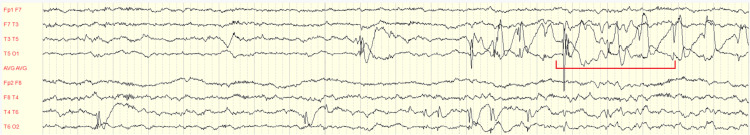
Paroxysmal activity of spike-wave accidents in the left temporal leads (T5), more frequent during sleep.

A few months later, another convulsive crisis occurred with moaning, hypertonia of the upper limbs, hypotonia of the remaining body, combined ocular deviation to the right and urinary incontinence. The EEG while being awake revealed paroxysmal activity of spike-wave accidents in left posterior temporal and right occipital leads (Figure 1). During sleep recording, basic electrogenesis was regularly structured, and the paroxysmal activity mentioned above became more frequent (Figure 2). The following conclusion was made: paroxysmal left posterior temporal and right occipital activity accentuated during sleep.

The child was diagnosed with SeLEAS and began maintenance treatment with carbamazepine. Furthermore, SOS treatment with rectal diazepam for seizures lasting >5 minutes was prescribed. A crisis calendar was provided to record every episode.

Psychological assessment revealed normal general intellectual functioning with vulnerability in the language area, which could compromise future learning skills.

She had an appointment with her family doctor, as part of a global health assessment, where parents mentioned their child´s learning difficulties. The family doctor made a referral to speech therapy sessions and booked an appointment six months later to check the child´s progress in language development and the frequency of epileptic crises.

## Discussion

This case report provides an opportunity to review the classification of epileptic syndromes, especially the most common in childhood.

The International League Against Epilepsy (ILAE) Classification of the Epilepsies was updated in 2017; it presents three levels of diagnosis: seizure type, epilepsy type and epilepsy syndrome.

The seizure type is the starting point of the epilepsy framework classification and is divided into focal onset, generalized onset, or unknown onset. The second level is epilepsy type, including generalized epilepsy, focal epilepsy, and a new category, combined generalized and focal epilepsy. An epilepsy syndrome incorporates seizure type, EEG and imaging features that occur together [6].

SeLEAS, formerly known as PS, is an idiopathic epilepsy syndrome with an excellent prognosis and normal findings on magnetic resonance imaging, characterized by a clinical ictal triad of nocturnal seizures, tonic deviation of the eyes and autonomic manifestations, including vomiting. Additionally, it is one of the most benign epilepsy syndromes regarding seizure frequency and remission. Cumulative results indicate that 40% of patients have a single seizure, 48% of patients have 2-5 seizures, and only 5% of patients have >10 seizures. Furthermore, 90% of patients achieve complete remission within 1-2 years of onset [7].

This syndrome can be extremely insidious and is often misdiagnosed, mainly because emetic and other autonomic manifestations are not recognized as seizures and may suggest nonepileptic conditions, such as atypical migraine, gastroenteritis, motion sickness, syncope, or sleep disorder. Moreover, prolonged and severe episodes may stimulate more severe diseases, such as encephalitis, intoxication, or even metabolic diseases. The main consequences of misdiagnosis are avoidable morbidity, erroneous treatments and costly hospital admissions [4,8].

In this case report, the first episode, only partially witnessed by parents, with non-specific and few post-ictal symptoms led to the misdiagnosis of gastroenteritis. Therefore, a rigorous objective examination with the characterization of the crisis and associated symptoms is important. Furthermore, epileptic syndromes should be considered in differential diagnosis of children with an episode of impaired state of consciousness.

Monitoring these children is crucial since depending on the frequency of epileptic crises and their consequences, they can lead to limitations in neurodevelopment, such as in language development.

A study of children with PS referred to the Kempenhaeghe Epilepsy Centre, a tertiary epilepsy centre in the Netherlands, found that these patients were eight months behind in arithmetic speed and 11 months behind in reading speed for the number of months in school. Additionally, behavioural questionnaires revealed significantly higher scores on reported internalizing behavioural problems [3,5,9].

## Conclusions

This case report illustrates a late presentation of a benign epileptic syndrome, SeLEAS, with an unusual course and neurocognitive sequelae. Since similar cases might occur, this case report highlights the importance of considering epileptic syndromes in the differential diagnosis of impaired state of consciousness in children.

Although PS is self-limited, children can have frequent seizure episodes and even negative consequences in neurodevelopment. This raises the need for an early accurate diagnosis, which is essential to begin maintenance treatment, prognosis, and adequate follow-up.
